# Consultation liaison to support efficient delivery of mental health care

**DOI:** 10.1192/bjo.2021.835

**Published:** 2021-06-18

**Authors:** Emma Davies, Mihaela Bucur

**Affiliations:** Sussex Partnership NHS Foundation Trust

## Abstract

**Aims:**

To study the impact of collaborative working, via consultation liaison, between Mental Health Liaison Practitioners (MHLPs) and Doctors within a secondary care mental health service. We hypothesise that this model of working may avoid unnecessary clinic appointments and waiting times, whilst providing patients with more efficient treatment.

**Background:**

Mental health services are stretched, understaffed and under-resourced. It is estimated that 75% of people with mental health problems in England may not get access to the treatment they need. We therefore need efficient and innovative ways for people who seek help to receive support. Good practice consultation liaison involves face to face contact between clinicians; treatment can be delivered by supporting primary care whilst reducing the burden of secondary care mental health services.

**Method:**

Regular 30-minute sessions within an Assessment and Treatment Service, between MHLPs and Doctors, at both Consultant and Trainee level, were coordinated. Patients assessed by MHLPs were discussed by opening a dialogue whereby further management was discussed across a multi-professional team. A record was created of all patients discussed and the outcome.

**Result:**

Number of MHLP/Doctor sessions: 10 across a six-month period.

Number of patients discussed: 17.

Medication advice provided for 16 patients. One patient required a referral for a clinic appointment.

For several patients, integrated working procured alternative care pathways and resources to be considered, to incorporate into individual treatment plans.

**Conclusion:**

Regular consultation liaison with MHLPs and Doctors is a model of working across the interface between primary care and specialist mental health services. It may provide patients with more efficient care, whilst avoiding unnecessary waiting times for clinic appointments. The consultation liaison working supported the development of an educative relationship between clinicians, with interprofessional learning. This is an example of an integrated and collaborative care model, whereby multi-professional working can provide efficient and effective treatment, whilst the support for the patient can remain in the primary care setting.

